# Chromosome comparison among five species of Neotropical cichlids of *Cichlasoma* and *Gymnogeophagus* (Perciformes)

**DOI:** 10.1590/1678-4685-GMB-2018-0383

**Published:** 2020-04-22

**Authors:** Larissa Bettin Pires, Mariana Campaner Usso, Lucia Giuliano-Caetano, Ana Lúcia Dias

**Affiliations:** 1 Universidade Estadual de Londrina, Centro de Ciências Biológicas, Departamento de Biologia Geral, Londrina, PR, Brazil.

**Keywords:** Chromosome banding, fish cytogenetics, karyotype diversification, ribosomal DNA

## Abstract

The genera *Cichlasoma* and *Gymnogeophagus* belong to the subfamily Cichlinae, the only one in Neotropical cichlids. *Cichlasoma dimerus*, *C. paranaense*, *C. portalegrense*, *Gymnogeophagus rhabdotus,* and *G. lacustri*s were collected at different points in the Paranapanema and Paraguay basins and the Lagoon of Patos hydrographic system. In addition to conventional analysis, CMA3 fluorochrome staining, and FISH with 18S rDNA probe were performed. All species had a diploid number equal to 48, with interand intraspecific differences in karyotype formulae. All species presented a single AgNOR site, except *G. rhabdotus* and the *C. paranaense* population of the Paranapanema River, which revealed more than one pair of nucleolar chromosomes. AgNORs were coincident to 18S rDNA and CMA3. Heterochromatin was distributed in the pericentromeric chromosomal regions and coincident with NORs. For the first time, this work shows cytogenetic data for *C. portalegrense*, *G. lacustris*, and *G. rhabdotus*. Although some results reinforce the idea of conservative chromosome evolution of 2n in Cichlinae, interspecific and populational variations observed confirm that chromosomal rearrangements affect the microstructural karyotype diversification in this group of fish.

## Introduction

Cichlidae represents the largest and most diverse family among Neotropical Perciformes, with about 1700 fish species (Eschmeyer and Fong, 2018). Based on morphological and molecular data, Smith *et al.* (2008) proposed that all Neotropical cichlids belong to a single subfamily, Cichlinae, as a monophyletic group. This subfamily is subdivided into seven tribes: Astronotini, Chaetobranchini, Cichlasomatini, Cichlini, Geophagini, Heroini, and Retroculini. The genera *Cichlasoma* and *Gymnogeophagus* belong to the Cichlasomatini and Geophagini tribes, respectively (Kullander, 2003). *Cichlasoma* presents a wide distribution, occurring in almost all Neotropical regions, from Mexico to the South of South America (Rican and Kullander, 2006). In contrast, *Gymnogeophagus* has a more restricted distribution, in which the majority of species is endemic to the coastal river drainage of Uruguay and southern Brazil, in the states of Rio Grande do Sul and Santa Catarina, with exception of *G. balzanii*, which presents a wider distribution (Reis and Malabarba, 1988).

Most of the species of Neotropical cichlids, approximately 60%, present a karyotype with 2n = 48, but a variation from 2n = 32 to 2n = 60 is observed, and chromosomal rearrangements have already been reported in the family (Feldberg *et al.*, 2003; Poletto *et al.*, 2010). Several cytogenetic analyses with the Cichlasomatini tribe show great chromosomal variation in this tribe (Feldberg *et al.*, 2003) in contrast with low ecomorphological diversity, compared with other tribes, such as Geophagini (López-Fernandes *et al.*, 2013), with few chromosomal data (Feldberg and Bertollo, 1984; Pires *et al.*, 2010; Paiz *et al.*, 2017). Hence, these tribes are of interest for cytogenetic studies.

Most cytogenetic studies on Neotropical cichlids are limited to the description of the karyotypic macrostructure (Thompson, 1979; Feldberg and Bertollo, 1985). In recent years, different classes of repetitive DNA have been used to better understand the karyotypic structure of Neotropical cichlids (Gross *et al.*, 2010; Poletto *et al.*, 2010). However, available information is restricted to a small number of species.

This work presents a comparative karyotype analysis of five species of cichlids: *Cichlasoma paranaense*, *C. dimerus*, *C. portalegrense*, *Gymnogeophagus rhabdotus*, and *G. lacustris*, using techniques of conventional and molecular chromosomal banding, and provides the first cytogenetic information for the last three species. The data presented are a contribution to a better understanding of the structure and karyotype evolution in this group of fish.

## Materials and Methods

The species of *Cichlasoma* and *Gymnogeophagus* were collected from different localities of the Paranapanema (PR/SP) and Paraguay/MS hydrographic basins and the hydrographic system Lagoon of Patos/RS (Table 1). The specimens were deposited in the Museum of Zoology at the State University of Londrina (MZUEL) under the voucher numbers: 3937 (*Cichlasoma paranaense* - Taquari), 3479 (*C. paranaense* - Paranapanema), 13128 (*C. dimerus*), 4860 (*C. portalegrense*), 20102 (*Gymnogeophagus rhabdotus*), and 20103 (*G. lacustris*). For convenience, different populations of *C. paranaense* were called population A (Taquari) and population B (Paranapanema), as shown in Table 1. The samples were collected with the permission of the Instituto Brasileiro do Meio Ambiente e dos Recursos Naturais Renováveis (IBAMA), protocol number 11399-1. We also obtained permission from the research ethics committee of the State University of Londrina (Animal Use Ethics number: CEUA 5579.2018.72).

**Table 1:** Collection sites and hydrographic basins of Cichlidae specimens analyzed. MS = Mato Grosso do Sul; PR = Paraná; RS = Rio Grande do Sul.

Species	Collection sites	Hydrographic basins	Number of individuals
*C. paranaense*	Taquari stream/PR 23º10’45.2’’S/50º56’30.9’’W	Paranapanema River-PR	4M,2F
*C. paranaense*	Paranapanema river/SP 22º42’30.3’’S /1º04’08.4’’W	Paranapanema River-PR	2M,2F
*C. dimerus*	Miranda river-MS 19°31’24.96”S/57°02’25.51”W	Paraguai River-MS	4M,6F
*C. portalegrense*	Estação Experimental Agronômica da UFRGS (30º5’38.38’’S 51º40’22.4’’W)	Laguna dos Patos/RS	5M,3F
*Gymnogeophagus rhabdotus*	Estação Experimental Agronômica da UFRGS (30º5’38.38’’S 51º40’22.4’’W)	Laguna dos Patos/RS	3M,3F
*G. lacustres*	Rondinha Lagoon (30º13’53.25’’S 50º15’15.17’’W)	Laguna dos Patos/RS	2M
	Total of individuals: 38		

M: male. F: female.

Mitotic chromosomes were obtained by direct preparation removing the anterior kidney according to Bertollo *et al.* (1978) and then stained with 5% Giemsa in phosphate buffer (pH 6.8). The morphology of the chromosomes was determined based on the ratio of arms, as proposed by Levan *et al.* (1964). For determination of the fundamental number (FN), the meta-submetacentric (m-sm) chromosomes were considered biarmed and the subtelo-acrocentric (st-a) uniarmed.

Silver nitrate staining revealed active nucleolus organizer regions (AgNORs) and was performed according to Howell and Black (1980). The distribution of constitutive heterochromatin was analyzed by Giemsa C-banding after treatments with 0.1 M HCl, Ba(OH)_2_, and 2 X SSC (Sumner, 1972). GCand AT-rich sites were detected with chromomycin A_3_ (CMA_3_) and 4’,6-diamino-2-phenylindole (DAPI) according to Schweizer (1980). Fluorescence *in situ* hybridization (FISH) was performed according to the protocol of Pinkel *et al.* (1986), with modifications according to Gouveia *et al.* (2013), using an 18S rDNA probe (Hatanaka and Galetti Jr, 2004). Finally, the slides were analyzed on an epifluorescence microscope (Leica DM2000), equipped with a digital camera. Metaphase images were captured using the Leica Application Suite version 3.1.0. (Leica Microsystems).

## Results

**Figure 1:**
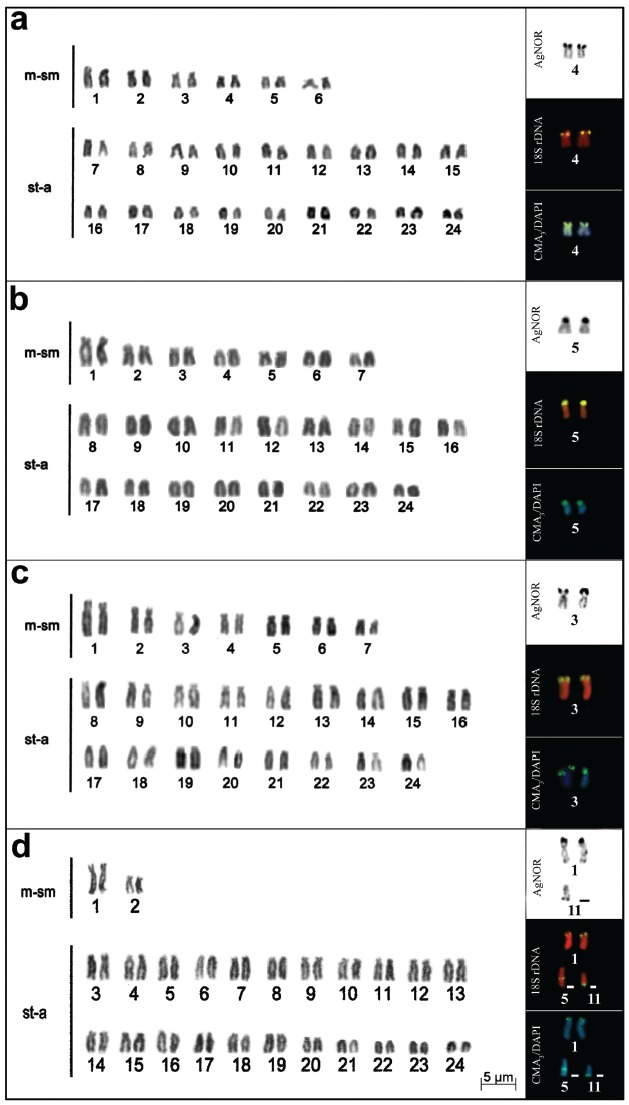
Karyotype and chromosome pairs with silver nitrate staining, FISH with 18S rDNA probe, and CMA_3_/DAPI in *Cichlasoma dimerus* (a), *C. portalegrense* (b), and *C. paranaense*, populations A (c) and B (d), respectively.

**Figure 2:**
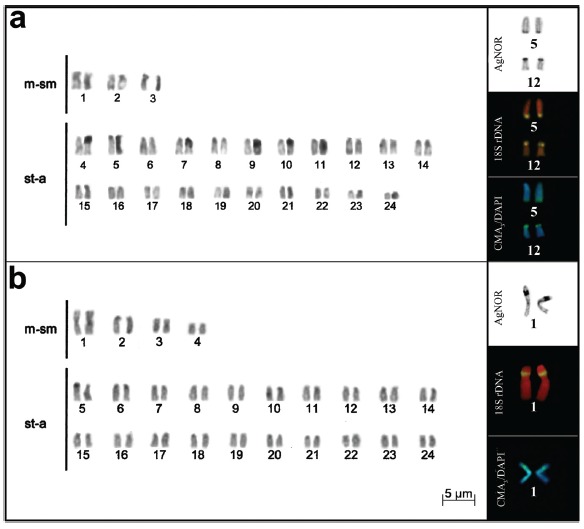
Karyotype and chromosome pairs with silver nitrate staining, FISH with 18S rDNA probe, and CMA_3_/DAPI in *Gymnogeophagus rhabdotus* .(a) and G. lacustris (b), respectively.

All specimens of *Cichlasoma* and *Gymnogeophagus* presented a diploid number (2n) equal to 48; however, different karyotype formulae were found: 12m-sm + 36st-a and a fundamental number (NF) equal to 60 for *Cichlasoma dimerus* (Figure 1a), 14m-sm + 34st-a (NF = 62) for *C. portalegrense* and population A of *C. paranaense* (Figures 1b and 1c, respectively) and 4m-sm + 44 st-a (NF = 52) for the population B of *C. paranaense* (Figure 1d). *Gymnogeophagus rhabdotus* showed 6m-sm + 42st-a (NF = 54), and *G. lacustris* 8m-sm + 40st-a (NF = 56) (Figures 2a and 2b, respectively). In the latter, an interstitial secondary constriction was identified in the short arm of the largest chromosomal pair, with small heteromorphism (Figure 2b, Table 2). No differences were observed between the karyotypes of males and females.AgNORs were located on a pair of chromosomes for all species, except for the population B of *C. paranaense* and *G. rhabdotus*, which showed three to four chromosomes bearing these regions (Figures 1 and 2, boxes). In the population B of *C. paranaense,* it was possible to observe a variation of two to three AgNORs in the terminal regions of the short arm of a submetacentric pair (pair 1) and the long arm of a subtelo-acrocentric chromosome (chromosome 11) (Figure 1d, box). In *Gymnogeophagus rhabdotus*, the AgNORs were located on st-a chromosomes: long arm of pair 5 and short arm of pair 12 (Figure 2a).

**Table 2:** Karyotype results for the species of *Cichlasoma* and *Gymnogeophagus* analyzed in this study: 2n = diploid number, FN = fundamental number, SC= secondary constriction, NORs = nucleolar organizer regions; CMA_3_ = chromomycin A_3_.

Species	Locality	2n	Karyotype formula	FN	SC	NORs	CMA_3_
*C. paranaense*	Taquari stream (PR) - population A	48	14 m-sm + 34 st-a	58	-	Single: par 3 (t)	par 3 (t)
	Paranapanema river (SP) - popula- tion B	48	4 m-sm + 44 st-a	58	-	Multiple: par 1 (t) crom 5 (i) e 11 (t)	par 1 (t) crom 5 (i) e 11 (t)
*C. portalegrense*	Estação Agronômica da UFRGS (RS)	48	14m+ 34 st-a	62	-	Single: par 5 (t)	par 5 (t)
*C.dimerus*	Miranda river (MS)	48	12m+ 36 st-a	60	-	Single: par 4 (t)	par 4 (t)
*G. rhabdotus*	Estação Agronômica da UFRGS (RS)	48	4m+2 sm+ 42 st-a	54	-	Multiple: par 5 (t) par 12 (t)	par 5 (t) par 12 (t)
*G. lacustris*	Rondinha lagoon (RS)	48	4m+4 sm+ 40 st-a	56	par 1 (i)	Single: par 1 (i)	par 1 (i)

The other species of *Cichlasoma*, including population A of *C. paranaense*, presented terminal AgNOR on the short arm of one pair of meta-submetacentric chromosomes (Figures 1a-c, boxes); in *G. lacustris* AgNOR was located interstitially on the short arm of the largest metacentric pair (Figure 2b). Staining with fluorochromes revealed CMA ^+^/DAPIcoincident with NORs in all species (Figures 1 and 2).

FISH with 18S rDNA probe demonstrated that *C. dimerus*, *C. portalegrense*, *C. paranaense* (population A), and *G. lacustris,* present two ribosomal cistrons corresponding to AgNORs (Figures 1a-c, and 2b, boxes). In the other two species, four ribosomal cistrons were observed: in pairs 5 and 12 in the terminal region of *G. rhabdotus* (Figure 2a, box), and in *C. paranaense* (population B) in the short arm of pair 1, in the long arm of chromosomes 5 and 11, and in interstitial and terminal regions, respectively (Figure 1d, box).

**Figure 3:**
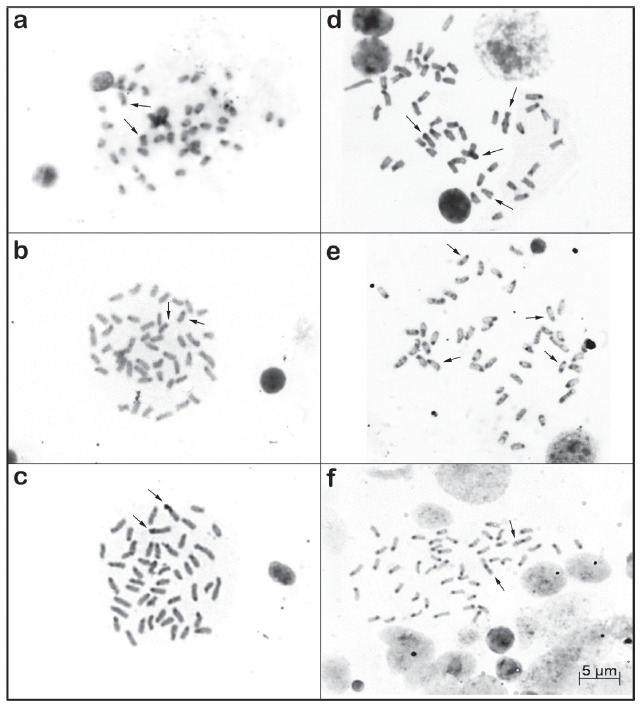
Somatic metaphases after C banding in *Cichlasoma dimerus* (a), *C. portalegrense* (b), *C. paranaense*, populations A (c) and B (d), *Gymnogeophagus rhabdotus* (e) and *G. lacustris* (f), respectively. The arrows indicate the NORs...

Heterochromatic regions were observed in the pericentromeric regions of the majority of chromosomes and associated with NORs in all species (Figure 3); *C. paranaense* also showed an interstitial marking on the long arm of a subtelo-acrocentric chromosome of pair 5 (Figure 3d) corresponding to NOR, and in *G. rhabdotus* terminal heterochromatic blocks were observed in some chromosomes (Figure 3e).

## Discussion

Despite conservation in diploid number, variations were found in the karyotype formulae of *C. dimerus* and *C. paranaense* (population B) in comparison to previously studied populations (Martins *et al.*, 1995; Feldberg *et al.*, 2003; Roncati *et al.*, 2007; Poletto *et al.*, 2010). Pericentric inversions seem to be the mechanism that predominantly contributed to these variations, since the diploid number was not altered, as observed by Thompson (1979), Feldberg *et al.* (2003), and Poletto *et al.* (2010) in other cichlid species. However, other rearrangement events cannot be ruled out in the family, as in *Tilapia mariae,* in which chromosomal fusion processes would explain the reduction of 2n to 40 chromosomes (Poletto *et al.*, 2010), and in *Symphysodon* species, where successive translocation events, fissions, and/or fusions would have contributed to the formation of the most highly derived karyotype in the Cichlidae family (2n = 60) (Mesquita *et al.*, 2008).

Recent studies show that the centromeres can be repositioned without any chromosomal rearrangement (Rocchi *et al.*, 2012). This phenomenon of centromere repositioning could explain the difference in the karyotype formulae between *C. paranaense* of the two localities, as also proposed by Schneider *et al.* (2013) for some species of cichlids.

Except for population B of *C. paranaense* and *G. rhabdotus*, which presented multiple NORs, all cichlids analyzed in the present study had only one nucleolar chromosomal pair, characterizing a single NOR system and confirming the ancestral condition proposed by Feldberg *et al.* (2003). However, differences in chromosome types and location of these sites were observed. These results are similar to those found in other species of *Cichlasoma* and *Gymnogeophagus*, such as C*. facetum* (Feldberg and Bertollo, 1985; Vicari *et al.*, 2006), *C. paranaense* (Martins *et al.*, 1995), and *G. labiatus* (Pires *et al.*, 2010), presenting only a variation in the identification of the carrier chromosome, or in metacentric (Martins *et al.*, 1995) or subteloacrocentric chromosomes (Vicari *et al.*, 2006), evidencing once again that chromosomal rearrangements are occurring in the group.

*Gymnogeophagus rhabdotus* presented two chromosomal pairs bearing ribosomal cistrons, an unusual pattern in the Geophaginae tribe, even though only few species were analyzed. However, there are reports of single NORs in *Geophagus brasiliensis*, *Gymnogeophagus gymnogenys*, and *Satanoperca acuticeps* (Brum *et al.*, 1998; Feldberg *et al.*, 2003; Pires *et al.*, 2010), and multiple NORs only in *Gymnogeophagus setequedas* (Paiz *et al.*, 2017). In population B of *C. paranaense*, a chromosomal pair and two non-homologous chromosomes (chromosomes 5 and 11) with ribosomal cistrons were observed; chromosome 5 had an interstitial signal, coincident with the heterochromatin, but not corresponding to AgNOR sites. The occurrence of 18S rDNA sites in non-homologous chromosomes and the location of these genes in the long arm are uncommon in *C. paranaense,* and may indicate a particular characteristic of this species and population. According to the literature, most sites are located on the short arm of the chromosomes, and can be of the m-sm group (Poletto *et al.*, 2010; Perazzo *et al.*, 2011), or the st-a group (Vicari *et al.*, 2006; Pires *et al.*, 2008; Gross *et al.*, 2010; Poletto *et al.*, 2010).

In the Geophagini and Cichlasomatini tribes, as in Cichlidae in general, the pattern of single NORs is the most common one (Poletto *et al.*, 2010), indicating that this characteristic can be considered plesiomorphic. Reports of multiple NORs, confirmed by FISH in cichlids, are scarce, and were reported in only seven species, including those described in this study: *Mesonauta festivus* (Poletto *et al.*, 2010), *Symphysodon aequifasciatus S. discus* and *S. haraldi* (Gross *et al.*, 2010), and *Gymnogeophagus setequedas* (Paiz *et al.*, 2017). It is worthy of note that four of these species of the genera *Mesonauta* and *Symphysodon* belong to the Heroini tribe, considered as derived within the subfamily Cichlinae. The NORs were CMA_3_ positive, rich in GC base pairs, as already shown in other species of Geophaginae and Cichlasomatinae by Loureiro *et al.* (2000), Vicari *et al.* (2006), and Pires *et al.* (2010).

The heterochromatin in the species of this study maintains the typical general distribution pattern found in cichlids, in pericentromeric and terminal regions, as observed in different species of *Cichlasoma* (Martins *et al.*, 1995; Vicari *et al.*, 2006; Roncati *et al.*, 2007) and *Gymnogeophagus* (Roncati *et al.*, 2007; Pires *et al.*, 2010), except for the population B of *C. paranaense,* which also pre sented a chromosome with interstitial marking.

The location of NORs in terminal regions may be the factor that facilitates the transposition of these sequences to other chromosomes through translocation events, as observed by Gross *et al.* (2010) in some species of *Symphysodon*, which could explain the origin of the interstitial ribosomal cistron found in only a large subtelo-acrocentric chromosome (chromosome 5). In addition, the association of heterochromatin and ribosomal sites may be related to the variability in location and number of the active NORs, a pattern commonly observed in Neotropical cichlids (Schneider *et al.*, 2013). Besides that, the differences between the populations may be due to their geographical isolation, so that this could facilitate the fixation of chromosomal rearrangements in the populations (Oliveira *et al.*, 1988), and possibly *C. paranaense* is a cryptic species.

The karyotype pattern observed in the species of this study reinforces the idea of a conservative diploid number in this group of fish. However, variations in karyotype formulae and location of NORs among the species and populations of *C. paranaense* confirm that chromosomal rearrangements are acting in the diversification of this group of fish.
